# Characterization of *Alternaria* Species Associated with Heart Rot of Pomegranate Fruit

**DOI:** 10.3390/jof7030172

**Published:** 2021-02-27

**Authors:** Francesco Aloi, Mario Riolo, Simona Marianna Sanzani, Annamaria Mincuzzi, Antonio Ippolito, Ilenia Siciliano, Antonella Pane, Maria Lodovica Gullino, Santa Olga Cacciola

**Affiliations:** 1 Department of Agriculture, Food and Environment, University of Catania, 95123 Catania, Italy; francesco.aloi@unipa.it (F.A.); mario.riolo@unirc.it (M.R.); 2 Department of Agricultural, Food and Forest Sciences, University of Palermo, 90128 Palermo, Italy; 3 Council for Agricultural Research and Agricultural Economy Analysis, Research Centre for Olive, Citrus and Tree Fruit–Rende CS (CREA- OFA), 87036 Rende, Italy; 4 Department of Agricultural Science, Mediterranean University of Reggio Calabria, 89122 Reggio Calabria, Italy; 5 CIHEAM Bari, Via Ceglie 9, 70010 Valenzano, Italy; sanzani@iamb.it; 6 Department of Soil, Plant, and Food Sciences, University of Bari Aldo Moro, Via Amendola 165/A, 70126 Bari, Italy; annamaria.mincuzzi@uniba.it (A.M.); antonio.ippolito@uniba.it (A.I.); 7 Agroinnova—Centre of Competence for the Innovation in the Agro-Environmental Sector, University of Turin, 10095 Turin, Italy; ilenia.sic@gmail.com (I.S.); marialodovica.gullino@unito.it (M.L.G.)

**Keywords:** *Alternaria alternata*, *Alternaria arborescens*, morphotypes, four-gene phylogeny, mycotoxins

## Abstract

This study was aimed at identifying *Alternaria* species associated with heart rot disease of pomegranate fruit in southern Italy and characterizing their mycotoxigenic profile. A total of 42 *Alternaria* isolates were characterized. They were obtained from pomegranate fruits with symptoms of heart rot sampled in Apulia and Sicily and grouped into six distinct morphotypes based on macro- and microscopic features. According to multigene phylogenetic analysis, including internal transcribed spacer (ITS), translation elongation factor 1-α (EF-1α), glyceraldehyde-3-phosphate dehydrogenase (GAPDH) and a SCAR marker (OPA10-2), 38 isolates of morphotypes 1 to 5 were identified as *Alternaria alternata*, while isolates of morphotype 6, all from Sicily, clustered within the *Alternaria arborescens* species complex. In particular, isolates of morphotype 1, the most numerous, clustered with the ex-type isolate of *A. alternata*, proving to belong to *A*. *alternata*. No difference in pathogenicity on pomegranate fruits was found between isolates of *A. alternata* and *A. arborescens* and among *A. alternata* isolates of different morphotypes. The toxigenic profile of isolates varied greatly: in vitro, all 42 isolates produced tenuazonic acid and most of them other mycotoxins, including alternariol, alternariol monomethyl ether, altenuene and tentoxin.

## 1. Introduction

In Italy, the commercial cultivation of pomegranate is rapidly expanding and has grown from around 130 ha in 2013 to 1234 ha in 2019 [1,2]. The most successful cultivar is Wonderful, originating from California and then introduced by Israeli in Italy, which is suitable for consumption as fresh fruit and fresh-cut products as well as for processing to produce juice. Moreover, several industrial and medical applications of pomegranate peel extracts, e.g., as food preservatives, are being envisaged, as pomegranate peel is rich in phenolic compounds, which are responsible for strong antioxidant and antimicrobial activity [3,4,5,6,7,8,9,10].

Currently, a major constraint of pomegranate commercial production is constituted by heart rot, an emerging disease caused by *Alternaria* spp. and reported from California, India and several Mediterranean countries, including Cyprus, Greece, Egypt, Israel and Italy [1,11,12,13,14,15]. The disease is also named Alternaria heart rot or black heart [16,17]. No precise estimation of losses caused yearly by heart rot to pomegranate production in Italy is available. According to the first report of the disease in Italy, its incidence in commercial orchards varies from 1 to 9% of fruits [1]. However, due to the difficulty in screening infected fruits on the basis of external symptoms, there are few chances to detect the presence of infected fruits, thus causing a serious value loss of the whole fruit stock.

Different *Alternaria* species were identified as causative agents of pomegranate heart rot on the basis of morphological characteristics and multilocus phylogenetic analyses [1,13,18,19]. Airborne spores of the pathogen are thought to cause flower infections [13,18]. Following fruit onset, infections can remain latent for most of the growing season until the establishment of favorable conditions [13]. Since the affected fruits are more prone to fall, one of the preventive agronomic practices is shaking the trees before harvesting [17]. Precise identification of the causative agent of heart rot is crucial for all aspects concerning the epidemiology and management of the disease. Unfortunately, the taxonomy of *Alternaria* is problematic and has undergone several revisions [20,21,22]. The difficulties in the identification of this genus at species level are related to the considerable morphological plasticity of most of the recognized *Alternaria* species. Historically, the identification of *Alternaria* spp. was based on morphological characteristics, including cultural features, size and shape of conidia and branching patterns of conidial chains [23,24]. Although the main sections of *Alternaria* can be differentiated using these features, this approach is not sufficient for distinguishing closely related species [25,26]. In the past, the production of host-specific toxins (HSTs) has been used to distinguish among species [27], but this criterion proved unreliable since HST biosynthetic gene clusters are located on small conditionally dispensable chromosomes, which can be lost or gained [28]. Molecular identification based on ribosomal DNA (rDNA), a genomic region typically used in fungal systematics, failed to differentiate small-spored *Alternaria* species [29,30,31]. Furthermore, the poor resolution obtained even using more variable genetic loci generated debate on which species should be kept within the *Alternaria* section *Alternaria* (i.e., *A. alternata*, *A. tenuissima*, *A. arborescens*, *A. mali* and *A. gaisen*), suggesting to combine *A. alternata* and *A. tenuissima* or to merge the latter two species with *A. arborescens* [20,25,26].

The analysis of secondary metabolites has also been proposed as a means to support species identification within the genus [22,32]. *Alternaria* is one of the major mycotoxigenic fungal genera [22,32,33,34,35,36,37,38,39]. Furthermore, it produces more than 70 phytotoxic metabolites, including host-specific toxins and non-host-specific toxins [40]. However, only a few mycotoxins (e.g., tenuazonic acid, alternariol, alternariol monomethyl ether) may be found in food and are of major toxicological concern. These toxins are suspected to exert both acute and chronic detrimental effects [41]. As a consequence, their presence as contaminants in pomegranate fruits and juice may represent a threat to human health.

This study aimed at the characterization of *Alternaria* isolates obtained from pomegranate fruit with symptoms of heart rot in southern Italy and at determining their mycotoxigenic profile.

## 2. Materials and Methods

### 2.1. Alternaria Isolates

Overall, 42 *Alternaria* isolates obtained from pomegranate fruit with symptoms of heart rot were included in this study (Table 1).

They were sourced from fruits of the three pomegranate cultivars Wonderful (Californian/Israeli origin), Mollar de Elche (Spanish origin) and Dente di Cavallo (Italian origin) [42,43], picked up in commercial pomegranate orchards in Apulia and Sicily from 2015 to 2016.

Isolates were preserved in the collection of the laboratory of Molecular Plant Pathology at the Department of Agriculture, Food and Environment (Di3A) of the University of Catania, Italy. Reference strains of *A. alternata* and *A. arborescens* from CBS-KNAW were included for comparison (Table 2).

### 2.2. Symptoms, Distribution and Incidence of the Disease

**Figure 1:**
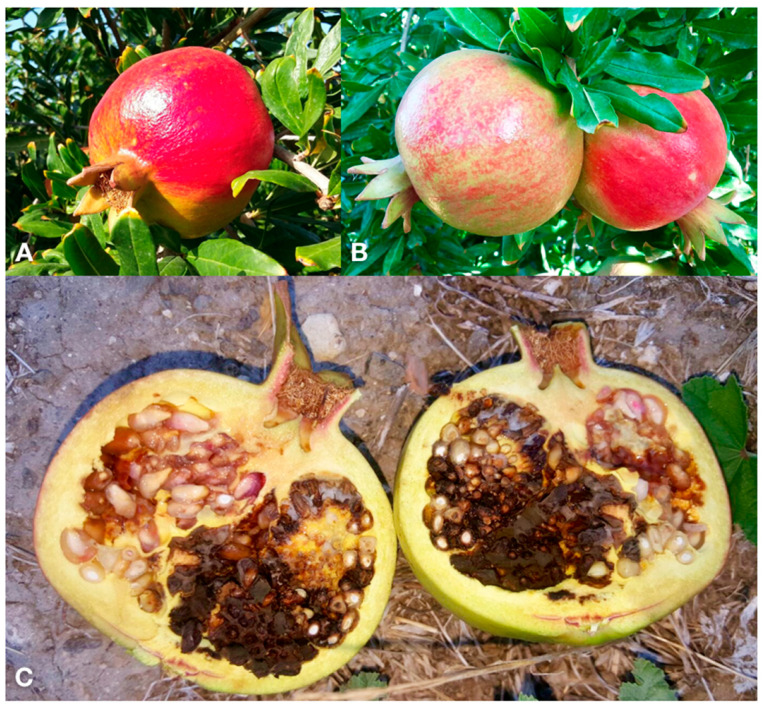
(**A,B**) External symptoms of heart rot in pomegranate fruit: dark-red discoloration and wrinkling of the peel. (**C**) Cut-open fruit of pomegranate with typical symptoms of heart rot. Note the dark brown mass of conidia produced by *Alternaria* sporulating on rotten arils within the fruit. Symptoms were restricted by membranes that separate the fruit compartments.

Infected fruits were characterized by a brown to black, soft to dry rot of the arils, visible when the fruit was cut open. Typically, the rot was confined to some aril compartments (Figure 1C) and did not affect the peel and compartment membranes (septa). The outer peel (epicarp), in correspondence of the internal rot, showed symptoms difficult to recognize, such as a dark-red discoloration and wrinkling of the peel (Figure 1A,B).

Heavily affected fruits were asymmetric and lighter in weight. The incidence of pomegranate fruits affected by heart rot in southern Italy was estimated to vary from 1 to 9% [1], although it might have been higher under favorable conditions, such as rainy and warm weather during flowering and early fruit development [44]. Heart rot is considered a serious postharvest problem due to the difficulty in recognizing affected fruits from external symptoms [1,13,17,44].

### 2.3. Morphological Characterization

**Figure 2:**
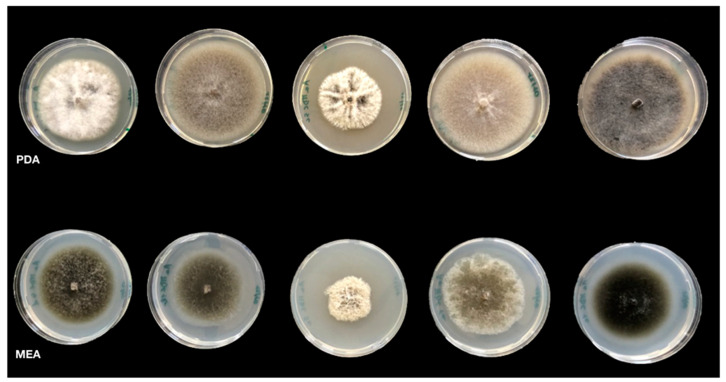
Growth pattern and colony morphology of isolates of *Alternaria* spp. obtained from pomegranate fruits with symptoms of heart rot collected from different Sicilian and Apulian orchards. From left to right: AaMDc1a, AaMP4, AaMRa1, AaMR4 and AaMDc5d. All the *Alternaria* isolated were grown on Potato Dextrose Agar (PDA) and Malt Extract Agar (MEA) culture media and incubated at 25 ± 1 °C for 7 days in the dark.

Isolates were grown in Petri dishes on Potato Dextrose Agar (PDA; Oxoid Ltd., Basingstoke, UK) and Malt Extract Agar (MEA; Sigma-Aldrich, Burlington, MA, USA), prepared according to CBS-KNAW Fungal Biodiversity Centre (Utrecht, The Netherlands) [45]. Dishes were incubated for 7 days at 25 ± 1 °C in the dark (Figure 2).

The macroscopic characteristics (color, margin, diameter and texture) of colonies were examined according to Pryor and Michailides [46], whereas microscopic features (conidium and conidiophore branch morphology) according to Simmons [24].

### 2.4. Molecular Characterization

Isolates were grown on PDA for 7 days at 25 ± 1 °C. Mycelium of each isolate was harvested with a sterile scalpel, and the genomic DNA was extracted using a PowerPlant^®^ Pro DNA isolation Kit (MO BIO Laboratories, Inc., Carlsbad, CA, USA), following the manufacturer’s protocol. The DNA was preserved at −20 °C. A multilocus approach was adopted to characterize and determine the phylogenetic allocation of the 42 isolates from pomegranate fruit.

Portions of four *Alternaria* barcoding genes/regions, i.e., internal transcribed spacer (ITS), translation elongation factor 1-α (EF-1α), glyceraldehyde-3-phosphate dehydrogenase (GAPDH) and one SCAR marker (OPA10–2), were sequenced [20]. The primers used for amplifying these genes/regions were ITS1/ITS4 for ITS [47], EF1-728F/EF1-986R for EF-1α [48], GPD1/GPD2 for GAPDH [49] and OPA10-2R/OPA10-2L for OPA10-2 [25] (Table 3).

PCR amplifications were performed on a GeneAmp PCR System 9700 (Applied Biosystems, Monza-Brianza, Italy). All PCR reactions were carried out by using Taq DNA polymerase recombinant (Invitrogen™) in a total volume of 25 μL containing PCR Buffer (1×), dNTP mix (0.2 mM), MgCl_2_ (1.5 mM), forward and reverse primers (0.5 μM each), Taq DNA Polymerase (1 U) and 1 μL of genomic DNA. Reaction conditions were 94 °C for 3 min followed by 35 cycles of 94 °C for 30 s, 55 °C (ITS region)/58 °C (EF-1α)/54 °C (GAPDH)/62 °C (OPA10-2) for 30 s, and 72 °C for 30 s, followed by an additional 10-min extension at 72 °C.

The amplicons were detected on 1% agarose gel, and purified products were sequenced with both forward and reverse primers by Macrogen Europe (Amsterdam, The Netherlands).

Sequences were analyzed by using FinchTV v.1.4.0 [50], and the consensus sequences were deposited in GenBank (Table 2).

For molecular identification, sequences obtained in the present study and validated sequences of CBS representative strains of species within *Alternaria* sect. *Alternaria*, were phylogenetically analyzed. Before analyses, the complete panel of reference sequences was tested utilizing Elim Dupes software [51] to delete multiple identical sequences. Identical reference sequences were included in the panel when representative of different *Alternaria* species [20]. Sequences were aligned using MUSCLE and introduced to MEGA6 for phylogenetic analysis with the Maximum Likelihood method using the Tamura–Nei model [52]. Analyses were performed with 1000 bootstrap replications. In order to maximize the effectiveness of the investigation into the genetic diversity among isolates obtained in the present study, the phylogenetic analysis was conducted using a combined dataset of all sequenced markers (ITS, EF-1α, GPDH and OPA10-2).

### 2.5. Pathogenicity Tests

The pathogenicity of two *A. arborescens* isolates (AaMDc1A and AaMDc1d) and six *A. alternata* isolates, one for each morphotype from 2 to 5 (AaMR2b, AaMR5b, AaMDc3a and AaMDc1a) plus two for morphotype 1 (M95 A2 and AaMP3, from Apulia and Sicily, respectively), was tested in a commercial farm at Lentini (Syracuse, Sicily) on pomegranate fruits ‘Wonderful’, using the injection method described by Luo et al. [14]. In detail, on 15 August 2020, a conidial suspension (5 µL of 2 × 10^5^ conidia·mL^−1^) was injected with a syringe into one side of pomegranate fruits (10 fruits per each isolate). Control fruits were injected with sterile distilled water. Ten weeks later, fruits were cut open longitudinally into two halves to observe heart rot symptoms.

### 2.6. Extraction and Analyses of Secondary Metabolites

Isolates were tested for production of secondary metabolites using a modified Czapek-Dox liquid medium: 10 g/L glucose, 0.162 g/L NH_4_NO_3_, 1.7 g/L KH_2_PO4, 0.85 g/L MgSO_4_, 0.425 g/L NaCl, 0.425 g/L KCl, 0.017 g/L FeSO_4_, 0.017 g/L ZnSO_4_ and 1.7 g/L yeast extract, pH 5.5. Cultures were inoculated with three mycelial plugs in 50 mL of medium. All cultures were performed in triplicate and incubated in the dark at 28 °C. After 8 days, cultures were filtered, and *Alternaria* toxins in the clear medium were extracted by liquid-liquid extraction. Each sample was adjusted to pH 2 with HCl, and an aliquot (5 mL) was transferred in a separating funnel. Ten milliliters of dichloromethane were added three times, and the mixture was shaken for 1 min, then, the dichloromethane extracts were collected in a flask. The final extract was evaporated to dryness in a rotary evaporator at 35 °C. The residue was dissolved in 1 mL of H_2_O/CH_3_OH 1:1 for the HPLC-MS/MS simultaneous detection of the main five *Alternaria* toxins. Analyses were carried out according to a previously validated method [35].

Standards of tenuazonic acid (TeA) copper salt from *A. alternata* (purity ≥ 98%), alternariol (AOH) from *Alternaria* spp. (purity ≥ 94%), alternariol monomethyl ether (AME) from *Alternaria alternata* (purity ≥ 98%), altenuene (ALT) from *Alternaria* spp. (purity ≥ 98%) and tentoxin (TEN) from *Alternaria tenuis* (purity 99%) were purchased from Sigma-Aldrich in crystallized form. A stock solution of 1 mg/mL and a working solution of 10 μg/mL were prepared in methanol for each molecule and kept at −20 °C. Standards for HPLC calibration and standards for addition experiment were prepared by diluting the working solution, and a calibration curve was built for each analyte. Good linearity was obtained for all analytes (*R*^2^ > 0.999). Recovery experiments were done spiking the matrix before extractions with a standard solution and the calculated recovery ranged between 80 and 100%.

## 3. Results

### 3.1. Morphological Characterization of Isolates

All 42 isolates were grouped according to macro- and microscopic features on PDA and MEA, along with reference strains of *A. alternata* and *A. arborescens* from CBS-KNAW. Higher variability in colony morphology was observed on PDA, as compared to MEA (Figure 2), allowing the differentiation of six morphotypes.

In particular, on PDA, twenty isolates (AaMMH7d, AaMDc5d, AaMP3, M24-BB1, AaMMH6b, AaMMH6a, AaMR9a, AaMR7, AaMR11, AaMDc5b, AaMMH7a, AaMMH7b, M103 A2-1, M109 3, M95 A2, AaMMH6d, AaMP7a, AaMP10, AaMMH6e and AaMP9) showed colonies that were flat, woolly, with colors ranging from brown to black and an average diameter of 65 mm. They produced dark brown conidia arranged in branched chains. Conidia appeared oval-ellipsoidal with 3–5 transverse septa. These features matched those of *A. alternata* ex-type reference strain CBS 916.96 (morphotype 1).

Nine isolates (AaMR2b, M80 B5, AaMP4, AaMR4, AaMR14a, AaMR14b, AaMP14b, AaMR12 and AaMP14a) showed greenish colonies with white margins. Conidia appeared elongated with a long-tapered beak. The characteristic sporulation pattern matched that of the reference strain of *A. alternata* (ex *A. tenuissima*) CBS 112252 (morphotype 2).

One isolate (AaMR5b) exhibited a colony that was pale brown, flat, granulated with undulating edges. Conidia appeared long and ellipsoidal with 1–3 transverse septa. The sporulation pattern resembled that of *A. alternata* (ex *A. limoniasperae*) CBS 102595 (morphotype 3).

Four isolates (AaMDc3a, AaMDc3b, AaMDc3c and AaMDc3d) exhibited a sporulation pattern resembling that of the reference strain of *A. alternata* (ex *A. citri*) CBS 102.47, with elliptical and subglobose conidia (morphotype 4).

Four isolates (AaMDc2a, AaMDc2b, AaMP6b and AaMR6b) exhibited wide and long conidia, with a sporulation pattern resembling that of *A. alternata* (ex *A. toxicogenica*) CBS 102600 (morphotype 5).

Four isolates (AaMDc1a, AaMRa1, AaMDc1d and AaMDc1b) showed colonies varying from greenish grey to brown, characterized by a lower growth rate (average diameter 45 mm after 7 days on PDA). Conidia appeared oval or ellipsoidal with 1–4 transverse septa and 1 or 2 longitudinal septa. They were borne by long primary conidiophores, occasionally presenting subterminal branches. These characteristics matched those of the reference strain for *A. arborescens* CBS 109730 (morphotype 6).

### 3.2. Molecular Characterization

**Figure 3:**
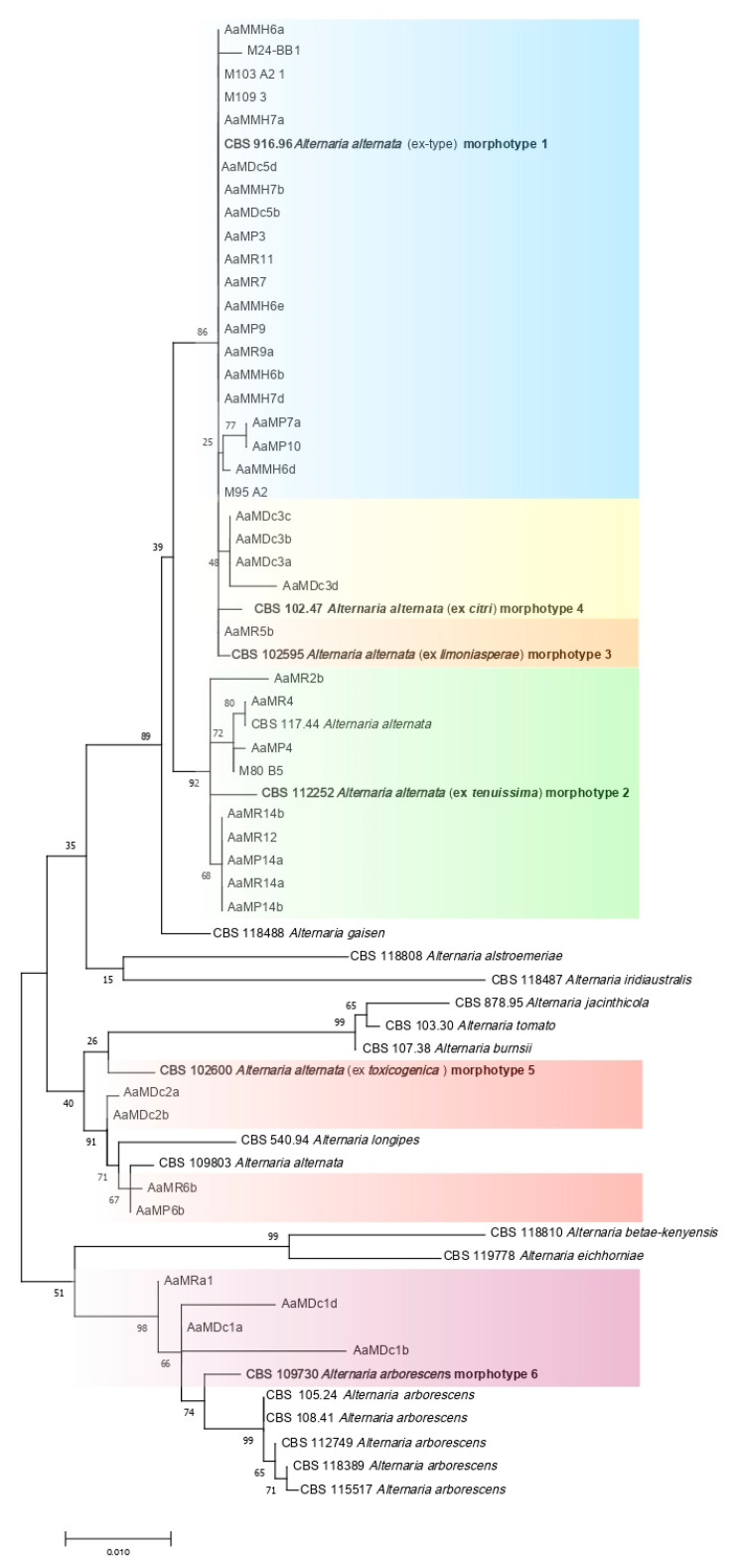
Internal transcribed spacer (ITS), glyceraldehyde-3-phosphate dehydrogenase (GAPDH), translation elongation factor 1-α (EF-1α) and one SCAR marker (OPA 10-2) multilocus phylogenetic tree developed using the Maximum Likelihood Method, based on the Tamura–Nei model. The tree with the greatest log likelihood (-3746.14) is shown. Relationships between the 42 isolates from pomegranate sourced in southern Italy and the CBS reference isolates of *Alternaria alternata*, *A. arborescens* (in bold CBS isolates used as reference for diverse morphotypes) and other *Alternaria* spp. The six morphotypes are highlighted in different colors.

*Alternaria* isolates from pomegranate sourced in southern Italy and reference isolates from CBS [20] were grouped on the basis of four-gene phylogeny, including ITS, EF-1α, GAPDH and OPA 10-2 sequences. According to this analysis, 38 out of the 42 isolates were associated with *A. alternata*. They were from both geographical sampling areas, Sicily and Apulia, and clustered with reference isolates of *A. alternata*, including the isolates CBS 916.96 (ex-type), and CBS 112252 (Figure 3). The *A. alternata* phylogentic group included morphotypes from 1 to 5. The most numerous phylogenetic subgroup, corresponding to morphotype 1, included the ex-type isolate of *A. alternata* CBS 916.96.

Conversely, four isolates, namely AaMDc1a, AaMRa1, AaMDc1d and AaMDc1b, all from Sicily, albeit from two different pomegranate cultivars, clustered within the *A. arborescens* species complex along with strains CBS 109730 from *Solanum lycopersicum*, CBS 105.24 from *S. tuberosum*, CBS 108.41 from wood, CBS 112749 from *Malus domestica*, CBS 118389 from *Pyrus pyrifolia* and CBS 115517 from *M. domestica*. This group of isolates corresponded to morphotype 6.

### 3.3. Pathogenicity Tests

**Figure 4:**
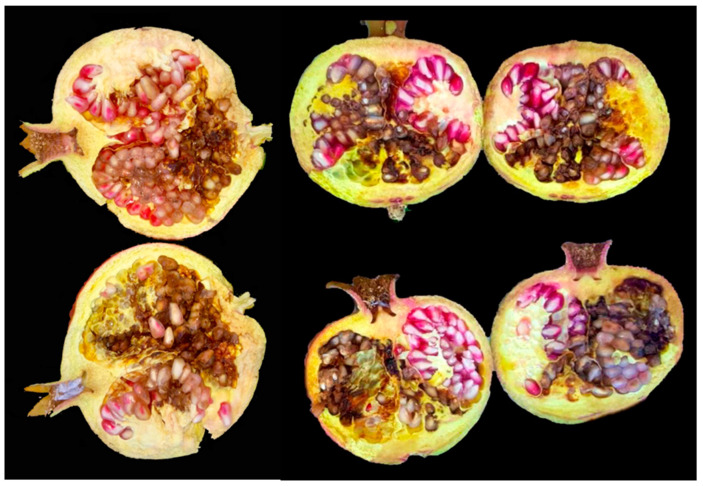
Symptoms of heart rot in pomegranate fruits artificially inoculated by injecting a suspension of *Alternaria* conidia, 10 weeks after inoculation.

All eight tested isolates of *A. alternata* and *A. arborescens* induced typical symptoms of heart rot on artificially inoculated pomegranate fruits and were reisolated from rotten arils. The proportion of symptomatic fruits for each isolate ranged from 88 to 100%, not to count inoculated fruits that dropped before harvesting (from one to two per each isolate). In each symptomatic fruit the rot extended to about half of the entire longitudinal section (Figure 4). No symptoms were observed on control fruit.

### 3.4. Analyses of Alternaria Mycotoxins

The toxigenic potential of the 42 *Alternaria* spp. isolates was investigated in vitro on liquid culture medium, and the concentration levels of the five mycotoxins extracted from culture filtrates are reported in Table 4. All isolates produced TeA, which was the prevalent mycotoxin found in culture extracts, with the only exception of isolates AaMR9a and AaMP9 of morphotype 1. Benzopyrone derivatives, AOH and AME, were produced by about 70% of the isolates. However, the amount of these two mycotoxins varied greatly among isolates and within species and morphotypes. The isolates that produced AOH also produced AME, but the concentrations of the two toxins in culture filtrates were not correlated. ALT, a third toxin in the class of dibenzo-α-pyrones, was quantified in less than 50% of samples; it was not detected in culture filtrates of isolates that did not produce AOH and AME. TEN, a cyclic tetrapeptide, was produced by 34 out of 42 *A. alternata* isolates, but its amount varied greatly, ranging from 3.09 to 2434 μg/L. None of the four strains of morphotype 6 (*A. arborescens*) tested produced TEN. Two isolates, AaMR14b and AaMP14a, both of morphotype 2 (*tenuissima*), produced only TeA. Overall, however, no correlation was found between the toxigenic profile of the isolates and their specific phylogenetic identity, morphotype and geographical origin.

## 4. Discussion

Results evidenced the complexity of *Alternaria* populations associated to heart rot of pomegranate fruits. According to the classification system proposed recently by Woudenberg et al. [20] for the small-spored *Alternaria* species in the *Alternaria* section *Alternaria*, the isolates recovered from symptomatic pomegranate fruit sampled in two major producing regions of southern Italy, Apulia and Sicily, were referred to *A. alternata* and *A. arborescens* species complex (AASC) [20], the former being by far the prevalent species. Despite the morphological and genetic variability, all tested isolates of both *A. alternata* and *A. arborescens* were pathogenic and induced typical symptoms of heart rot in pomegranate fruit artificially inoculated by injecting separately a conidial suspension of these two fungi into the fruit. These results indicate that *A. alternata* and *A. arborescens* are responsible for pomegranate heart rot disease in southern Italy. According to the available information, this is the first report of *A. arborescens* as a pathogen of pomegranate in Italy. Both species are known as plant pathogens and mycotoxin producers on a wide range of host plants [32,53]. Due to their growth even at low temperature, these *Alternaria* species are also responsible for spoilage of fruits and processed plant products during refrigerated transport and storage [54].

In agreement with a previous study aimed at identifying *Alternaria* species associated to brown spot of tangerines in southern Italy [19], *A. alternata* isolates recovered from pomegranate fruits showed great morphological and molecular variability and were separated into six morphotypes corresponding to distinct albeit phylogenetically related clusters. Our results confirmed previous findings of other authors in California showing that diverse species of *Alternaria* are associated to heart rot of pomegranate [14,18]. Although in California heart rot is regarded as a minor disease of pomegranate, the correct identification of *Alternaria* species associated to this disease is considered a crucial aspect, as *A. gaisen*, included in the section *Alternaria* and closely related to *A. arborescens*, is a quarantine pathogen, and its presence might impose export restrictions [14]. In the present study, multilocus phylogenetic analysis based on four gene regions ITS, EF-1α, GPDH and OPA 10-2, clearly separated *A. alternata*, *A. arborescens* and *A. gaisen*. Whereas, neither ITS nor EF-1α and GPDH, alone or in combination, were able to discriminate these three species. Consistently with the results of this study aimed at characterizing the diversity of *Alternaria* species associated to heart rot of pomegranate, *A. alternata* and *A. arborescens* were the species associated to the brown fruit rot of tangerines and mandarins in southern Italy and California, and also in these cases, *A. alternata* was the prevalent species [19,55]. It was demonstrated that the virulence of the *Alternaria* isolates recovered from citrus was positively correlated with the expression level of the ACTT1 and ACTT1 genes encoding for phytotoxins ACTT1 and ACTT2, respectively [19]. Several *formae speciales* of *A. alternata* infecting fruit and vegetable crops and producing host-specific toxins are currently recognized, including *A. alternata* f. sp. *lycopersici* producing AAL-toxins and causing necrotic lesions on tomato, *A. alternata* f. sp. *mali* producing the AM toxin, *A. alternata* f. sp. *fragariae* producing the AF-toxin, and *A. alternata* f. sp. *citri* with two pathotypes, i.e., pathotype rough lemon for isolates producing the ACR-toxin, and pathotype tangerine for isolates producing the ACT-toxins [39,56]. No f. sp. or pathotype of *A. alternata* have been identified so far within the *A. alternata* populations associated to pomegranate fruits.

In standard laboratory conditions, isolates of both *A. alternata* and *A. arborescens* recovered from pomegranate fruits with symptoms of heart rot were able to produce mycotoxins, mainly tenuazonic acid (TeA), a tetramic acid derivative, and to a lesser extent alternariol (AOH), alternariol monomethyl ether (AME) and altenuene (ALT), in the structural group of dibenzopyrone derivatives. The majority of *A. alternata* also produced the cyclic tetrapeptide tentoxin (TEN), whereas none of the tested *A. arborescens* isolates produced this metabolite. The results of the present study are consistent with previous reports indicating that small-spored species in the section *Alternaria* (*A. tenuissima*, *A. arborescens* and *A. alternata* species groups) share a common secondary metabolite profile, but only a small proportion of *A. arboresecns* isolates are able to produce TEN [57,58]. TeA, AOH, AME and TEN have a broad host range [40] and in pomegranate heart rot disease they might act as virulence factors [59]. Some of these non-host-specific toxins, such as TeA, AOH and AME, have been reported to induce harmful effects in mammals [60]. In vitro tests provided clear evidence of genotoxicity or acute toxicity in animals or rodent cells of AOH, AME, ALT and TeA [41]. Moldy arils of fresh pomegranate fruits affected by heart rot are usually removed by the consumer, as they are not appropriate for human consumption. However, toxins can move from the rotten part to the surrounding tissues or processed fruit products. This may occur more frequently in *Alternaria* diseases that go unnoticed, as symptoms are internal, such as pomegranate heart rot. Moreover, current industrial processing methods do not exclude the risk of juice contamination by *Alternaria* toxins [33,60,61]. As a consequence, the occurrence of heart rot on pomegranate fruits and of *Alternaria* toxins as contaminants in juice may be a risk factor for consumer health. In a comprehensive study of EFSA (European Food Safety Authority) aimed at investigating the distribution of *Alternaria* toxins across the food categories and groups, AOH, AME, ALT, TeA and TEN were the toxins occurring most commonly in fruit and vegetable juices [41]. In this study the ability of *Alternaria* species associated to heart rot of pomegranate to produce toxins has been investigated for the first time in Europe.

## 5. Conclusions

The diversity of *Alternaria* responsible for heart rot of pomegranate in southern Italy, encompassing ubiquitous and polyphagous species with both a saprophytic and pathogenic lifestyle, like *A. alternata* and *A. arborescens*, and the ability of all identified *Alternaria* genotypes to induce disease in artificially wound inoculated fruits would suggest this is a complex disease. Generally speaking, environmental factors and host plant susceptibility usually have a key role as disease determinants of this type of diseases. The factors favoring the susceptibility of pomegranate fruits to infection by *A. alternata* and the onset of Alternaria heart rot in commercial pomegranate orchards in Israel have been investigated, but results are not yet conclusive [62]. Our study also highlights another aspect of Alternaria heart rot of pomegranate, namely the toxigenic potential of *Alternaria* species associated with the disease, which has practical implications for the juice industry and the EU regulations of the limits of mycotoxins in foods. The actual risk and level of contamination of pomegranate juice by *Alternaria* toxins during the industrial processing have not yet been quantified and deserve to be thoroughly investigated. Tools to rapidly screen internally rotten pomegranate fruits during postharvest processing, thus preventing the risk of contamination by mycotoxins, are being envisaged [17]. A practical method to identify fruits affected by heart rot might be to shake the trees before harvest, as internally rotten fruits are more prone to drop, whereas fungicide treatments proved to be scarcely effective in preventing the disease [13].

## Figures and Tables

**Table 1:** *Alternaria* isolates from pomegranate fruits characterized in this study, their geographical origins and accession numbers of their internal transcribed spacer (ITS), translation elongation factor 1-α (EF-1α), glyceraldehyde-3-phosphate dehydrogenase (GAPDH) and a SCAR marker (OPA 10-2) sequences in GenBank.

Isolate	Morphotype	Location	Host, Cultivar	Accession Numbers			
				ITS	EF-1α	GAPDH	OPA 10-2
AaMR7	1	Italy, Sicily	*Punica granatum* cv. Wonderful	MW580732	MW585113	MW590491	MW590533
AaMP7a	1	Italy, Sicily	*Punica granatum* cv. Wonderful	MW580733	MW585114	MW590492	MW590534
AaMP10	1	Italy, Sicily	*Punica granatum* cv. Wonderful	MW580734	MW585115	MW590493	MW590535
AaMR11	1	Italy, Sicily	*Punica granatum* cv. Wonderful	MW580735	MW585116	MW590494	MW590536
AaMMH6e	1	Italy, Sicily	*Punica granatum* cv. Mollar de Elche	MW580743	MW585121	MW590502	MW590544
AaMP3	1	Italy, Sicily	*Punica granatum* cv. Wonderful	MW580745	MW585123	MW590504	MW590546
AaMR9a	1	Italy, Sicily	*Punica granatum* cv. Wonderful	MW580747	MW585125	MW590506	MW590548
AaMP9	1	Italy, Sicily	*Punica granatum* cv. Wonderful	MW580748	MW585126	MW590507	MW590549
AaMDc5b	1	Italy, Sicily	*Punica granatum* cv. Dente di cavallo	MW580754	MW585132	MW590513	MW590555
AaMDc5d	1	Italy, Sicily	*Punica granatum* cv. Dente di cavallo	MW580755	MW585133	MW590514	MW590556
AaMMH6b	1	Italy, Sicily	*Punica granatum* cv. Mollar de Elche	MW580756	MW585134	MW590515	MW590557
AaMMH7a	1	Italy, Sicily	*Punica granatum* cv. Mollar de Elche	MW580757	MW585135	MW590516	MW590558
AaMMH7d	1	Italy, Sicily	*Punica granatum* cv. Mollar de Elche	MW580758	MW585136	MW590517	MW590559
AaMMH6a	1	Italy, Sicily	*Punica granatum* cv. Dente di cavallo	MW580763	MW585140	MW590522	MW590564
AaMMH6d	1	Italy, Sicily	*Punica granatum* cv. Mollar de Elche	MW580764	MW585141	MW590523	MW590565
AaMMH7b	1	Italy, Sicily	*Punica granatum* cv. Mollar de Elche	MW580765	MW585142	MW590524	MW590566
M24-BB1	1	Italy, Apulia	*Punica granatum* cv. Dente di cavallo	MW580768	MW585145	MW590527	MW590569
M95 A2	1	Italy, Apulia	*Punica granatum* cv. Wonderful	MW580770	MW585147	MW590529	MW590571
M103 A2-1	1	Italy, Apulia	*Punica granatum* cv. Wonderful	MW580771	MW585148	MW590530	MW590572
M109 3	1	Italy, Apulia	*Punica granatum* cv. Wonderful	MW580772	MW585149	MW590531	MW590573
AaMR4	2	Italy, Sicily	*Punica granatum* cv. Wonderful	MW580739	MW585117	MW590498	MW590540
AaMR12	2	Italy, Sicily	*Punica granatum* cv. Wonderful	MW580742	MW585120	MW590501	MW590543
AaMR2b	2	Italy, Sicily	*Punica granatum* cv. Wonderful	MW580744	MW585122	MW590503	MW590545
AaMP4	2	Italy, Sicily	*Punica granatum* cv. Wonderful	MW580746	MW585124	MW590505	MW590547
AaMR14b	2	Italy, Sicily	*Punica granatum* cv. Wonderful	MW580760	MW585137	MW590519	MW590561
AaMP14a	2	Italy, Sicily	*Punica granatum* cv. Wonderful	MW580761	MW585138	MW590520	MW590562
AaMR14a	2	Italy, Sicily	*Punica granatum* cv. Wonderful	MW580766	MW585143	MW590525	MW590567
AaMP14b	2	Italy, Sicily	*Punica granatum* cv. Wonderful	MW580767	MW585144	MW590526	MW590568
M80 B5	2	Italy, Apulia	*Punica granatum* cv. Wonderful	MW580769	MW585146	MW590528	MW590570
AaMR5b	3	Italy, Sicily	*Punica granatum* cv. Wonderful	MW580731	MW585112	MW590490	MW590532
AaMDc3a	4	Italy, Sicily	*Punica granatum* cv. Dente di cavallo	MW580751	MW585129	MW590510	MW590552
AaMDc3b	4	Italy, Sicily	*Punica granatum* cv. Dente di cavallo	MW580752	MW585130	MW590511	MW590553
AaMDc3c	4	Italy, Sicily	*Punica granatum* cv. Dente di cavallo	MW580753	MW585131	MW590512	MW590554
AaMDc3d	4	Italy, Sicily	*Punica granatum* cv. Wonderful	MW580762	MW585139	MW590521	MW590563
AaMR6b	5	Italy, Sicily	*Punica granatum* cv. Wonderful	MW580740	MW585118	MW590499	MW590541
AaMP6b	5	Italy, Sicily	*Punica granatum* cv. Wonderful	MW580741	MW585119	MW590500	MW590542
AaMDc2a	5	Italy, Sicily	*Punica granatum* cv. Dente di cavallo	MW580749	MW585127	MW590508	MW590550
AaMDc2b	5	Italy, Sicily	*Punica granatum* cv. Dente di cavallo	MW580750	MW585128	MW590509	MW590551
AaMDc1a	6	Italy, Sicily	*Punica granatum* cv. Dente di cavallo	MW580736	MW585150	MW590495	MW590537
AaMDc1b	6	Italy, Sicily	*Punica granatum* cv. Dente di cavallo	MW580737	MW585151	MW590496	MW590538
AaMDc1d	6	Italy, Sicily	*Punica granatum* cv. Dente di cavallo	MW580738	MW585152	MW590497	MW590539
AaMRa1	6	Italy, Sicily	*Punica granatum* cv. Mollar de Elche	MW580759	MW585153	MW590518	MW590560

**Table 2:** GenBank accession numbers of sequences of the *Alternaria* spp. isolates of different country and host origins used as references in phylogenetic analyses.

Species	Isolate	Country	Host	Accession Numbers ^a^			
				ITS	EF-1α	GPDH	OPA 10-2
*Alternaria alternata* (ex *A. citri*)	CBS 102.47	USA	*Citrus sinensis*	KP124304	KP125080	KP124161	KP124610
*Alternaria alternata* (ex-type)	CBS 916.96	India	*Ara* *chis hypogaea*	AF347031	KC584634	AY278808	KP124632
*Alternaria alternata* (ex *A. limoniasperae*)	CBS 102595	USA	*Citrus jambhiri*	FJ266476	KC584666	AY562411	KP124636
*Alternaria alternata* (ex *A. tenuissima*)	CBS 112252	-	*-*	KP124340	KP125116	KP124194	KP124650
*Alternaria alternata* (ex *A. godetiae*)	CBS 117.44	Denmark	*Godetia* sp.	KP124303	KP125079	KP124160	KP124609
*Alternaria gaisen*	CBS 118488	Japan	*Pyrus pyrifolia*	KP124427	KP124278	KP125206	KP124743
*Alternaria alstroemeriae*	CBS 118808	USA	*Alstroemeria* sp.	KP124296	KP125071	KP124153	KP124601
*Alternaria iridiaustralis*	CBS 118487	Australia	*Iris* sp.	KP124436	KP125215	KP124285	KP124752
*Alternaria jacinthicola*	CBS 878.95	Mauritius	*Arachis hypogaea*	KP124437	KP125216	KP124286	KP124753
*Alternaria tomato*	CBS 103.30	-	*Solanum lycopersicum*	KP124445	KP125224	KP124294	KP124762
*Alternaria burnsii*	CBS 107.38	India	*Cuminum cyminum*	KP124420	JQ646305	KP125198	KP124734
*Alternaria alternata* (ex *A. toxicogenica*)	CBS 102600	USA	*Citrus reticulata*	KP124331	KP125107	KP124186	KP124640
*Alternaria longipes*	CBS 540.94	USA	*Nicotiana tabacum*	AY278835	KC584667	AY278811	KP124758
*Alternaria alternata*	CBS 109803	Germany	human skin	KP124336	KP125112	KP124190	KP124645
*Alternaria betae-kenyensis*	CBS 118810	Kenya	*Beta vulgaris* var. *cicla*	KP124419	KP125197	KP124270	KP124733
*Alternaria eichhorniae*	CBS 119778	Indonesia	*Eichhornia crassipes*	KP124426	KP125205	KP124277	KP124741
*Alternaria arborescens*	CBS 109730	USA	*Solanum lycopersicum*	KP124399	KP125177	KP124251	KP124713
*Alternaria arborescens*	CBS 105.24	-	*Solanum tuberosum*	KP124393	KP125171	KP124245	KP124706
*Alternaria arborescens*	CBS 108.41	-	wood	KP124394	KP125172	KP124246	KP124707
*Alternaria arborescens*	CBS 112749	South Africa	*Malus domestica*	KP124401	KP125179	KP124253	KP124715
*Alternaria arborescens*	CBS 118389	Japan	*Pyrus pyrifolia*	KP124407	KP125185	KP124259	KP124721
*Alternaria arborescens*	CBS 115517	South Africa	*Malus domestica*	KP124404	KP125182	KP124256	KP124718

^a^ source [20].

**Table 3:** Primers used in this study and PCR conditions.

Primer	Primer DNA Sequence	PCR Conditions	Reference
ITS1	5’ TCC GTA GGT GAA CCT GCG G 3′	94 °C for 3 min; 94 °C for 30 s, 55 °C for 30 s, 72 °C for 30 s for 35 cycles and final extension at 72 °C for 10 min	[47]
ITS4	5’ GCT GCG TTC TTC ATC GAT GC 3′		
EF1-728F	5’ CAT CGA GAA GTT CGA GAA GG 3′	94 °C for 3 min; 94 °C for 30 s, 58 °C for 30 s, 72 °C for 30 s for 35 cycles and final extension at 72 °C for 10 min	[48]
EF1-986R	5’ TAC TTG AAG GAA CCC TTA CC 3′		
GPD1	5’ CAA CGG CTT CGG TCG CAT TG 3′	94 °C for 3 min; 94 °C for 30 s, 54 °C for 30 s, 72 °C for 30 s for 35 cycles and final extension at 72 °C for 10 min	[49]
GPD2	5’ GCC AAG CAG TTG GTT GTG C 3′		
OPA 10-2R	5’ GAT TCG CAG CAG GGA AAC TA 3′	94 °C for 3 min; 94 °C for 30 s, 62 °C for 30 s, 72 °C for 30 s for 35 cycles and final extension at 72 °C for 10 min	[25]
OPA 10-2L	5’ TCG CAG TAA GAC ACA TTC TAC G 3′		

**Table 4:** In vitro mycotoxin production [µg/mL] by *Alternaria* isolates after eight days of incubation at 28 ± 1 °C in the dark.

Isolate	Morphotype	Origin	Mycotoxin Concentration (μg/mL) ^a^				
			TeA	AOH	AME	ALT	TEN
AaMR7	1	Italy, Sicily	5133 ± 561	9.141 ± 1.46	2.094 ± 0.22	9.177 ± 1.32	167.5 ± 31.9
AaMP7a	1	Italy, Sicily	1555 ± 139	41.44 ± 7.22	26.56 ± 4.01	307.8 ± 34.7	303.2 ± 13.6
AaMP10	1	Italy, Sicily	1066 ± 125	n.d. ^b^	n.d.	n.d.	91.85 ± 1.76
AaMR11	1	Italy, Sicily	1889 ± 146	38.73 ± 3.95	23.98 ± 8.38	131.41 ± 13.5	214.4 ± 13.1
AaMMH6e	1	Italy, Sicily	279.4 ± 44.6	5.619 ± 0.44	4.735 ± 0.42	n.d.	1.378 ± 0.12
AaMP3	1	Italy, Sicily	495.6 ± 50.3	n.d.	n.d.	n.d.	3.16 ± 0.25
AaMR9a	1	Italy, Sicily	25.61 ± 0.48	n.d.	n.d.	n.d.	30.77 ± 2.28
AaMP9	1	Italy, Sicily	24.13 ± 2.02	7.734 ± 0.17	4.462 ± 0.18	17.98 ± 0.79	34.05 ± 4.67
AaMDc5b	1	Italy, Sicily	839.2 ± 65.7	n.d.	n.d.	n.d.	12.99 ± 2.02
AaMDc5d	1	Italy, Sicily	3146 ± 256	26.50 ± 0.32	12.34 ± 1.65	83.59 ± 2.33	40.80 ± 4.32
AaMMH6b	1	Italy, Sicily	786.8 ± 111	6.604 ± 0.29	2.451 ± 0.40	8.236 ± 1.31	n.d.
AaMMH7a	1	Italy, Sicily	2799 ± 132	55.49 ± 3.16	14.41 ± 1.21	36.82 ± 1.57	13.77 ± 0.72
AaMMH7d	1	Italy, Sicily	1004 ± 67.9	4.010 ± 1.14	3.978 ± 0.72	3.464 ± 0.32	1.295 ± 0.01
AaMMH6a	1	Italy, Sicily	1606 ± 168	57.24 ± 5.39	24.06 ± 0.35	44.33 ± 4.31	19.37 ± 2.13
AaMMH6d	1	Italy, Sicily	1553 ± 70.8	83.28 ± 4.30	22.71 ± 2.29	17.08 ± 0.54	3.455 ± 0.58
AaMMH7b	1	Italy, Sicily	2189 ± 149	11.64 ± 0.20	4.932 ± 0.05	n.d.	31.82 ± 0.25
M24-BB1	1	Italy, Apulia	4204 ± 650	222.3 ± 0.14	472.7 ± 1.21	n.d.	1957 ± 2.67
M95 A2	1	Italy, Apulia	5204 ± 233	161.2 ± 0.25	407.3 ± 1.97	n.d.	2434 ± 2.78
M103 A2-1	1	Italy, Apulia	1267 ± 198	547.7 ± 0.73	604.2 ± 0.60	n.d.	1655 ± 28.8
M109 3	1	Italy, Apulia	3258 ± 7.78	384.9 ± 0.09	196.5 ± 0.36	n.d.	n.d.
AaMR4	2	Italy, Sicily	1950 ± 56.5	23.82 ± 1.77	24.12 ± 4.32	n.d.	87.62 ± 4.76
AaMR12	2	Italy, Sicily	1117 ± 20.9	n.d.	n.d.	n.d.	20.80 ± 0.31
AaMR2b	2	Italy, Sicily	1177 ± 44.6	3.922 ± 0.64	2.209 ± 0.38	6.019 ± 0.27	3.09 ± 0.86
AaMP4	2	Italy, Sicily	789.3 ± 113	n.d.	n.d.	n.d.	15.19 ± 1.13
AaMR14b	2	Italy, Sicily	952.9 ± 0.34	n.d.	n.d.	n.d.	n.d.
AaMP14a	2	Italy, Sicily	488.2 ± 22.3	n.d.	n.d.	n.d.	n.d.
AaMR14a	2	Italy, Sicily	1169 ± 33.8	n.d.	n.d.	n.d.	3.542 ± 0.66
AaMP14b	2	Italy, Sicily	9227 ± 904	44.87 ± 2.87	6.603 ± 0.27	n.d.	5.986 ± 0.59
M80 B5	2	Italy, Apulia	2987 ± 1.09	39.15 ± 0.55	73.27 ± 0.68	n.d.	8621 ± 37.1
AaMR5b	3	Italy, Sicily	1264 ± 73.0	18.29 ± 1.63	23.59 ± 1.36	112.5 ± 2.51	153.2 ± 15.4
AaMDc3a	4	Italy, Sicily	1762 ± 38.6	37.64 ± 1.16	4.853 ± 0.01	17.97 ± 1.06	51.69 ± 5.91
AaMDc3b	4	Italy, Sicily	2887 ± 195	89.64 ± 2.97	10.77 ± 0.10	7.465 ± 0.28	27.94 ± 2.72
AaMDc3c	4	Italy, Sicily	2336 ± 154	n.d.	n.d.	n.d.	64.26 ± 2.55
AaMDc3d	4	Italy, Sicily	2532 ± 214	32.55 ± 0.60	4.905 ± 0.28	9.409 ± 0.40	77.48 ± 7.63
AaMR6b	5	Italy, Sicily	1460 ± 83.6	n.d.	n.d.	n.d.	235.7 ± 38.1
AaMP6b	5	Italy, Sicily	1115 ± 24.9	1.848 ± 0.01	4.928 ± 0.43	94.28 ± 8.51	201.4 ± 20.3
AaMDc2a	5	Italy, Sicily	2028 ± 92.3	n.d.	n.d.	n.d.	116.1 ± 6.53
AaMDc2b	5	Italy, Sicily	1840 ± 159	n.d.	n.d.	n.d.	51.42 ± 0.19
AaMDc1a	6	Italy, Sicily	2769 ± 207	56.18 ± 6.82	7.050 ± 1.71	425.97 ± 33.2	n.d.
AaMDc1b	6	Italy, Sicily	3918 ± 473	18.04 ± 2.17	4.074 ± 0.04	43.23 ± 0.92	n.d.
AaMDc1d	6	Italy, Sicily	1680 ± 171	33.12 ± 4.62	2.984 ± 1.26	67.54 ± 2.91	n.d.
AaMRa1	6	Italy, Sicily	2666 ± 19.5	76.98 ± 1.37	9.974 ± 1.29	384.92 ± 42.3	n.d.

^a^ Means ± standard error of three independent biological experiments consisting of three technical replicates each. ^b^ n.d.= not detected.

## Data Availability

Not applicable.
